# Integrating rare disease management in public health programs in India: exploring the potential of National Health Mission

**DOI:** 10.1186/s13023-022-02194-z

**Published:** 2022-02-10

**Authors:** Mohua Chakraborty Choudhury, Pragya Chaube

**Affiliations:** DST Center for Policy Research, Indian Institute of Science, Bengaluru, India

**Keywords:** National Health Mission, Rare diseases, RMNCH + A, RBSK, JSSK, Public health, National Policy for Rare Diseases 2021

## Abstract

Rare diseases (RD) are conditions that affect a small number of people and hence do not get the focus on government health priorities in a resource-constrained setting such as India. Therefore, it is essential to focus on strengthening and utilizing the existing public health framework for the optimal usage of healthcare resources. In this regard, National Health Mission (NHM) is one of the crucial programs initiated by the government of India to address the health needs of the under-served. As Phase 1 of the NHM moves towards completion, we explored the Reproductive, Maternal, Newborn, Child, and Adolescent Health (RMNCH + A) program under NHM to assess their potential and limitations to aid RD care. We found that some of the disease-prevention initiatives of NHM address certain RDs and can easily be expanded to manage many such preventable RDs. In addition, NHM programs can provide a unique epidemiological data repository to strengthen the National Rare Disease Registry. These programs can also play important role in providing a continuum of care for many RDs that need lifelong management. However, existing programs have a limited scope to provide specialized RD-related treatments, which is better served in a more focused system. Thus, considering RDs in the design of the existing programs may help RD management better through prevention, data collection, and providing a continuum of care.

**Supplementary Information:**

The online version contains supplementary material available at 10.1186/s13023-022-02194-z.

## Introduction

Rare diseases (RD) are a heterogeneous group of distinct and often debilitating diseases characterized by low prevalence in a population. The World Health Organization defines them as the conditions that affect one or less in 1000 people. Each country, however, has a subjective definition of RDs based on population prevalence and healthcare resources. Despite the low prevalence of individual RDs, collectively, they form a significant burden on public healthcare systems. By an estimation, globally the combined population prevalence of RDs is 3.5–5.9% at any given time [1]. This translates to 263–446 million people worldwide affected by RDs [1]. Children disproportionately account for 50–70% of the diagnosed RD cases [2]. Of the 6172 RDs in the Orphanet database, 70% have pediatric-onset [1]. Congenital anomalies form a significant group of RDs and are the fifth leading cause of perinatal mortality worldwide [3]. RDs are a substantial cause of pediatric hospital admissions too, resulting in the mortality of one-third of children born with diseases before their fifth birthday [2]. Those who do survive would likely live throughout their remaining lives with impairment and disabilities caused by the disease. A review of 351 disorders associated with handicaps and disabilities indicated that ¾ of such diseases with survival beyond infancy affected the ability to function normally in everyday life [4]. Naturally, prevention and management of neonatal and childhood disorders are essential components of RD management.

India does not yet have an official definition of RD. There is a massive lack of awareness about RDs among healthcare professionals, the public, policymakers and industry [5]. Many of the cases often remain undiagnosed, and those who do receive a diagnosis, are often not well recorded [5]. Also, due to the lack of prevalence data, the actual burden of RDs is not known. Nevertheless, India is expected to have a considerable burden of RDs due to its large population size, high birth rate, pervasive endogamy, high consanguinity rate, and other economic and sociocultural factors [6]. Extrapolation from global data shows that there could be around 70–96 million people living with RDs at any given time in India [5]. Additionally, child health indicators suggest that a large population could be affected by some form of RDs [7]. India accounts for a quarter of global neonatal deaths and the largest share of congenital anomalies per live birth [8]. An estimated 1.7 million birth defects in India account for 9.6% of newborn deaths [6, 9]. The census report of 2011 stated that 78,64,636 children are living with disabilities in India, constituting 1.7% of the total child population [10]. Every year approximately 10,000–15,000 children are born with a blood disorder [11]. Further, the growing body of evidence for individual rare diseases in ad-hoc/hospital-based studies [12–16] suggests that RDs are a significant public healthcare challenge in India and need immediate attention.

Nonetheless, RDs did not feature in any government agenda or health policy of the country until recently. In 2021, driven by a long-standing movement by patient advocacy groups, the Ministry of Health and Family Welfare (MoHFW), Government of India released the revised National Policy for Rare Diseases (NPRD) 2021. NPRD 2021 acknowledges the alarming crisis of RDs in the country. It also emphasizes the need for a public health surveillance system to bridge the knowledge gaps in epidemiological data, research, and awareness, which are obstacles in forming an effective RD management strategy. The policy aims to lower incidences of RDs by preventing the birth of children with RDs and puts forward recommendations for strengthening infrastructure to support and treat RD patients. The policy, however, also acknowledges that public health resources are limited in India and resources that can be channelized solely for RD management are scarce. Therefore, optimally utilizing the existing public healthcare programs to aid RD management becomes necessary.

For efficient utilization of resources, it becomes imperative to understand the utility, expandability, and limitations of the existing public healthcare resources in RD management. In the quasi-federal structure of governance in India, healthcare is the responsibility of the State/Union Territory (UT) governments. Each state/UT has its own healthcare system and resources and is responsible for the successful implementation of health policies formulated either by the state government or the federal/central government. However, the Central government is equally accountable for health governance under Article 21 (Right to Life) [17], and MoHFW is charged with national health policies and programs. A significant program under MoHFW is the National Health Mission (NHM), phase 1 of which was launched in 2013 with the objective to provide “universal access to equitable, affordable & quality health care services that are accountable and responsive to people's needs.” NHM provides an umbrella under which various standalone health programs, targeting different health components, can be integrated for efficient results. A vital thrust area under NHM is Reproductive, Maternal, Newborn, Child, and Adolescent health (RMNCH + A) which majorly aims to prevent preventable maternal and child mortality, thereby aligning with the aim of NPRD 2021 which prioritizes RD prevention. Further, through an office memorandum by MoHFW, matters pertaining to rare genetic disorders were brought under the purview of NHM [18]. Therefore, NHM is a critical nationwide program that can aid and enable RD management.

Despite the obvious importance, there has been minimal discussion on integrating RD management in public health programs in India. To the best of our knowledge, no previous study has explored the potential of any public health programs in India for providing RD care. This paper explores various NHM programs that can play an important role in RD screening and management. With the launch of phase 2 of NHM in view, we have tried to map the potential areas in RD care that can be served by the existing programs and areas that need attention through RD-focused programs.

### Features of NHM with potential for RD management

An effective RD management requires prevention, early diagnostics, timely intervention, rehabilitation of patients, and an efficient data driven public health surveillance system. Here, we discuss some of the key NHM programs under RMNCH + A that have the potential to cater to these RD management strategies as listed in Table 1 (please see Additional file 1 for further details).

**Table 1:** A list of National Health Mission’s RMNCH + A programs that aid or can potentially aid different aspects of RD management strategies

RD management strategies	Public health components	NHM programs
Primary prevention	Reproductive health	Family planning
	Maternal health	Pradhan Mantri Surakshit Matritva Abhiyan (PMSMA)
		Janani Shishu Suraksha Karyakram (JSSK)/Janani Suraksha Yojana (JSY)
Secondary prevention	Newborn health	Rashtriya Bal Suraksha Karyakram (RBSK)
	Child health	Rashtriya Bal Suraksha Karyakram (RBSK)
		Home-Based Young Care (HBYC)
Diagnosis	Newborn health	Rashtriya Bal Suraksha Karyakram (RBSK)
	Child health	
Treatment	Maternal health	Janani Shishu Suraksha Karyakram (JSSK)
	Newborn health	Facility-based newborn and childcare units (SNCU)
		Rashtriya Bal Suraksha Karyakram (RBSK)
	Child health	
Rehabilitation	Child health	Rashtriya Bal Suraksha Karyakram (RBSK)
Data collection and surveillance	Maternal health	MDSR/MCTS/RCH portals
	Newborn health	Rashtriya Bal Suraksha Karyakram (RBSK)/SNCU
	Child health	Rashtriya Bal Suraksha Karyakram (RBSK)
		Home-Based Young Care (HBYC)

Here we have used the official names of the programs which are mostly in Indian languages. However English translation of these names are provided in Additional file 2

Further details on NHM and discussed RMNCH + A programs can be found in Additional file 3.

### Primary prevention

Most RDs do not have an approved treatment, and for those that have treatment, prohibitive cost makes them unaffordable for Indian patients [19]. Therefore, it is essential to identify causal factors and plan systematic prevention strategies. RDs can be caused by various factors including genetic, environmental, exposure to teratogens (infections or toxic agents), maternal health, nutritional deficiencies, or unknown factors [20, 21]. Eighty percent of childhood RDs have a genetic origin [1, 19]. Thus, the primary prevention strategy of childhood RDs needs to integrate prevention mechanisms in existing prenatal and postpartum health programs.

Primary reproductive health care is an essential public health service and a cornerstone to RD management. Information and counselling on sexual reproductive health and family planning are important parts of the RMNCH + A strategy. These programs target to address complications at birth by preventive care (such as antenatal check-ups, birth preparedness), skilled care at birth, early detection of risk (with the use of partographs), appropriate and timely management of obstetric complications, and postnatal care. NHM Pregnancy Testing Kits are supplied under the brand name Nishchay to all the sub-centers and through community health workers such as Accredited Social Health Activist (ASHA). This can enable early detection of pregnancies, which is the first step towards early registration, and timely antenatal care [22]. Early detection of pregnancies is essential to monitor cases with a high risk of RDs, especially in families with a history of an RD. Furthermore, under the Family Planning program, counselling is provided on matters which are known to be critical factors for RD such as mother’s age, spacing between different pregnancies, etc. [19, 22].

Launched in 2011, the NHM program Janani Shishu Suraksha Karyakram (JSSK) is primarily aimed at promoting institutionalized deliveries by eliminating out-of-pocket expenses. The services provided under JSSK encompass the entire duration of pregnancy, including the crucial antenatal care period during which embryos are formed and are most susceptible to congenital anomalies. A major focus is on preventing infections in mothers during pregnancy or soon after. In addition, women are counseled on nutritional diets and provided drugs and supplements such as folic, iron acid, and iodine during antenatal, intranatal and postnatal care up to 6 weeks. The intake of micro-and macro-nutrients during pregnancy and post-partum period has been known to reduce the instances of neural tube defects, congenital hypothyroidism, anemia, stunting, and severe acute malnutrition in infants [20, 21]. Infections and metabolic conditions such as malnutrition are some of the known teratogens and such low-cost primary prevention strategies can potentially reduce or eliminate some of the risk factors [20, 21]. The program also facilitates access to public health institutions for the treatment of all sick newborns for the first 30 days of their lives. JSSK, thus, becomes an important program to monitor pregnancy, birth and prevent congenital anomalies.

Pradhan Mantri Surakshit Matritva Abhiyan (PMSMA), launched in 2016, supports pregnant mothers during the 2nd and 3rd trimester of pregnancy by detecting high-risk pregnancies and providing special antenatal services at government hospitals. Under this initiative, technologies such as ultrasound have been efficiently used in the gestational period to detect structural anomalies that can provide the family and relatives with more information and choices [23]. Families with previous history of an RD may be categorized under high-risk pregnancies. This provides an opportunity to the family to avail timely multidisciplinary maternal–fetal intervention and an option to terminate the pregnancy in extreme cases [23].

### Secondary prevention: screening and early identification

The secondary prevention strategies aim to identify a condition at an early stage, preferably before the onset of symptoms, allowing for early intervention. For many RDs timely intervention can contain the disease from progressing to a debilitating stage and prevent permanent disabilities. Newborn screening is one such preventive strategy which has been successfully implemented to manage or avoid the consequences of treatable RDs in children in many countries [21].

Rashtriya Bal Swasthya Karyakram (RBSK) is NHM’s flagship program for child health and screening that aims to improve the survival outcomes of children born with congenital anomalies through early identification and intervention. Under the RBSK program, children aged 0–18 years of age are screened for 30 diseases and childhood conditions grouped under the 4D’s viz: **D**efects at birth, **D**eficiencies, **D**iseases, **D**evelopment delays including disability. The newborn screening is undertaken by a healthcare professional at the delivery facility. It involves checking for vital signs and a thorough head-to-toe physical examination for visible birth defects. This comprehensive screening approach is essential as many RDs present symptoms across multiple organs. And this often impedes correct diagnosis when there is a lack of communication between different specialists treating each problem.

RBSK also allows for the early identification of diseases that manifest later in childhood. This is facilitated through child health monitoring for the children aged 6 weeks to 6 years at Anganwadi centers and 6 years to 18 years at government or government-aided schools. The monitoring is performed by mobile health teams at Anganwadi centers twice a year via physical examination and other non-invasive methods for developmental delays, disabilities, deficiencies, and malnutrition. Anganwadi centres are public health units in rural areas that provide basic child health services. Mobile health team monitors school children through questionnaires and clinical examinations once a year. Other important parameters, such as weight loss, signs of depression, fatigue, and drop in school performance are also considered for adolescents. The children identified under RBSK are referred to District Early Intervention Centres (DEIC) for further diagnosis and follow-up.

In addition to RBSK, different NHM initiatives incentivizes ASHAs to visit newborns up to 45 days after their birth under Home Based Newborn Care (HBNC) program and till 15 months from birth under Home-Based Care for Young Child (HBYC) program. The purpose of HBNC is to record the health data of the infant and mother and promote immunization. Under HBYC, children are assessed for nutrition, health, development, sanitation, and hygiene. ASHAs can refer a sick child to health facilities for management of complications. While these programs are primarily designed to focus on common childhood diseases, they could also be important for babies born with birth defects and RDs. For instance, each district hospital and sub-district hospital has been mandated to have Special Newborn Care Units (SNCU) to provide critical care for sick newborns. According to studies, 20% of the children discharged from SNCU develop developmental disabilities at a later age [24]. Through the HBYC program, quarterly home visits and monitoring of the children discharged from SNCU are made possible for early detection and intervention.

### Diagnosis

In India, on average, diagnosis of RDs takes about 7 years or more after the onset of symptoms. And in many cases, it remains undiagnosed [5]. There is an additional burden on the patients to travel to healthcare professionals with different expertise depending on different symptoms arising from multi-organ defects [21]. The RBSK program can potentially reduce this complexity. Neonates and children identified and referred to DEIC can avail multidisciplinary approaches to diagnostics under a single roof. The core services available at DEIC include medical, dental, psychological, cognitive, speech and audiology, vision, and psycho-social services, among others. Although these core services are essential for the early identification of diseases, DEICs are severely limited in human- and infrastructural-resources for exact diagnosis of RDs [24]. However, DEICs provide service coordination and referrals to appropriate tertiary healthcare services for further diagnosis, treatment, and management [9]. DEICs, therefore, despite being limited in their capacities to provide exact diagnosis, are integral to identify potential cases and integrate them in the RD management system through referrals and cross-referrals.

### Treatment and management

Among all the globally identified RDs, only 5% are treatable [19]. Most RDs do not have an approved treatment and patients usually receive non-targeted functional therapies for their symptoms. In the absence of epidemiological data, NPRD 2021 identifies three groups of disorders as RDs based on their treatment:

1. Group 1: diseases that require a single-time curative treatment; for example, organ transplant or Hematopoietic stem cell treatment.
2. Group 2: diseases that require long-term/lifelong treatment and have relatively lower cost. This group includes diseases that require dietary management, such as phenylketonuria, and diseases that require specific drugs or hormonal therapies.
3. Group 3: diseases that require cost-prohibitive lifelong treatment. Specific treatments are available under this category, however, there may be limited literature on the outcomes of the treatment.

Different NHM programs that provide treatment services to reduce mortality and morbidity in neonates and children may be expanded for the treatment and management of childhood RDs. Under the Facility-based newborn initiative by NHM, different levels of newborn care facilities are mandated. Newborn Care Corners functions at all delivery points to provide essential newborn care, whereas Newborn Stabilization Units are present at all community health centers/first referral units for management of selected newborn conditions and stabilization of newborns before referrals to secondary/tertiary healthcare facilities. In addition, each state/UT is mandated to have an SNCU within each district to support complicated pregnancies. This initiative not only strengthens newborn health infrastructure, but also provide an opportunity to support neonates born with congenital anomalies or some other RD-related complications. Further, under RBSK program treatment is also available for—(1) neural tube defects (2) cleft lips and palate (3) down syndrome (4) congenital cataract (5) congenital deafness (6) congenital heart disease (7) developmental dysplasia (8) club foot (9) retinopathy of prematurity [9]. Few other conditions are managed under JSSK [25]. These NHM programs, RBSK in particular, provide an opportunity to expand in their scope to include and treat Group 1 disorders that require corrective surgery for rare anatomical disorders and Group 2 disorders that may be addressed via lifestyle/dietary changes.

It is, however, important to reiterate that the NHM programs, in their current forms, have limited potential to provide treatment and management to RD patients. Only a handful of RDs maybe treated under RBSK and JSSK. Moreover, diseases in Group 1 requiring post-operative care, diseases in Group 2 needing lifelong therapies, and diseases in Group 3, are outside the purview of these programs. The majority of RDs are lifelong conditions requiring long-term advanced support and resources, whereas NHM provides support to neonatal and child health up to 18 years of age only.

### Rehabilitation and supplementary services

The financial burden of RD treatment and management has immense societal impact and huge implications on the individual patient and their family. Under the Janani Suraksha Yojana program, institutional delivery is promoted through cash entitlements to pregnant mothers [26]. Under JSSK, antenatal care, institutional care, and care for newborns (till the first 30 days of birth) are provided for free [25]. Under RBSK, diagnostics, referral, transport, and other services are provided free of cost at DEIC for children and students diagnosed with 4D health conditions [9]. The provision to remove out-of-pocket expenses from early prevention strategies, transportation, follow-ups, and early diagnosis are an asset to the developing nation and the RD community. However, the scope of NHM programs to cover RD diagnosis and treatment financing is very limited until now.

Being diagnosed with a RD often means living with a RD. RBSK provides rehabilitation opportunities to patients diagnosed with 4D by liaising with other ministries. The patients may receive disability certificates through DEIC managers, which would make them eligible for disability scholarships and pensions. Under Sarv Shiksha Abhiyaan (Indian government initiative aimed at universalization of elementary education; please see Additional file 2), children suffering from a 4D condition can avail inclusive or home-based education from special needs tutors on a needs basis. Support is provided to patients suffering from lifelong disorders using a family-centered approach that helps the patients in their everyday lives and activities. If RBSK may be expanded to RDs, the social services would give RD patients and their families a chance at a normal life.

### Data collection and surveillance

The epidemiological data in RDs include determining the prevalence, incidence, distribution, natural history, and causal factors [21]. This data helps define RDs in the country’s context and assess their burden, thereby guiding policy decisions in terms of resources allocation [21]. Further, such data provide clinical and medical knowledge for better diagnostics and treatment of the RDs, and facilitates innovation and technological advancements in therapeutics and related research [21].

A major handicap in RD management in India is the lack of epidemiological data. To address this gap, the Indian Council of Medical Research and the NPRD had envisaged a hospital-based National RD Registry in 2017. However, as it is not functional yet, data collection through the different NHM programs presents a unique opportunity to build a comprehensive database to survey childhood disorders, including RDs [21].

Programs such as JSY and JSSK have increased institutional deliveries, leading to additional data generation on maternal and newborn health status. Further, there is a mandate to collect and record various maternal and child health data by SNCUs, DEICs, and ASHAs. These data are stored and managed in different NHM-databases such as Maternal Death Surveillance and Response (MDSR)— to record instances of maternal deaths and causes; National Reproductive and Child Health (RCH) portal—to capture information on all RCH related services, including family planning, maternal health, child health, and immunization [27, 28].

## Discussion

Here, we explored the existing National Health Mission’s RMNCH + A programs from the lens of RD management strategies. The RMNCH + A initiatives cater to RD prevention through effective family planning, minimizing environmental components during pregnancy that contribute to birth defects, early detection of high-risk pregnancies, and integration of deliveries in the public health monitoring system. The early screening and identification of potential cases are further aided through child health programs such as RBSK and HBNC. The existing programs, however, are restricted in their scope to provide exact diagnosis, treatment, and management to RD patients. Further, implementation of these programs has faced limitations in terms of lack of infrastructure and equipment, lack of skilled human resources, lack of capacity building, short mapping of resources for referral, lack of  public private partnership models, etc. [24]. Additionally, few of the key white spaces which should be considered to strengthened and expand the scope of NHM programs to cater to RDs are discussed below:

### Awareness and medical education

Awareness and targeted medical education programs on RDs would engage with society, enabling them to seek professional help on time, take preventive actions and destigmatize RDs. Communities should be acquainted with RDs through sustained awareness campaigns using different channels such as social media, national programs on raising awareness on genetic conditions, etc. Integrating RD training and awareness programs with the existing medical education system would broaden the reach of RD knowledge within the medical community. India's apex body for training under NHM, the National Institute of Health and Family Welfare, could be a valuable resource in this regard. It focuses on public health education, the development of skills in public health management, and all training needs of the health care providers. This initiative can also empower grassroots community-level workers such as ASHAs to raise awareness of RDs. The ASHAs can also identify families with a history of RDs and provide premarital, post-marital, and preconception counseling—the core services as specified in NPRD 2021 to prevent the births of children with rare genetic disorders [21].

### Role of primary healthcare providers

Primary healthcare providers (PHP) play a vital role in enhancing the quality of life of patients and families living with a rare disease by making appropriate referrals to specialists, helping to coordinate care, and assisting patients in obtaining the proper support. They are the first point of contact and play a crucial role in identifying cases and early diagnosis. However, PHPs face unique challenges in RDs due to diagnostic delays and a lack of information, expertise, and treatment options for most RDs. Moreover, PHPs are overburdened in India with many patients and a skewed doctor-to-patient ratio. The focus should be given to make PHPs aware of RDs, training, and equipping them with proper referral services to Centres of Excellence (CoE) of RDs. In the long run, they could work with the patients and disease experts and enable a better continuum of care. This will be particularly useful for patients based far away from the CoEs. Although NPRD identifies DEICs as the point of reference to CoEs, engagement of PHPs from both public and private sectors in the identification, referral, and management process is essential.

### Referral services

Referral services play a vital role in RD treatment and management. For referral services, the onus is on the States/UT to map their healthcare resources and provide them to DEIC. This may not be restricted to the public sector, but the private sector may be included too. For certain RDs, it may be possible that a State/UT may not have enough resources and expertise, and proper management is available only at specific CoE/private or public healthcare providers in the country. It is, therefore, essential to map healthcare resources for such RDs at a national level and referral to apt services made available at DEIC for such ultra-rare cases. A proper referral mechanism for inter-state communication and telemedicine facilities should be developed for RD patients.

### Genetic diagnosis

Genetic testing and counseling are crucial in RD screening, diagnostics, and management. While presymptomatic genetic testing is a norm in several developed countries worldwide, implementing such a system in a developing country like India may not be as cost-efficient. By an estimate, the cost of screening all babies born in a year for a single congenital disease in India would be about 1.2% of the entire country’s health budget [29]. Therefore, instead of universal genetic screening and counseling, the focus could be targeted high-risk pregnancies. The lack of genetics laboratories in the States/UT has been acknowledged in NPRD 2021, and the formation of at least one medical genetics department in medical colleges per State/UT has been directed. NPRD 2021 further recommends that CoEs and National Inherited Diseases Administration Kendras be involved in providing genetics services and capacity building. Furthering the aim towards capacity building in medical professionals at Community Health Centres/Primary Health Centers (PHC), the Department of Biotechnology has implemented the pilot program Unique Methods of Management and Treatment of Inherited Disorders in the aspirational districts (please see Additional files 2 and 3 for details) [30]. These different initiatives aiming at under a single umbrella of NHM for the most efficient utilization of resources. Further, avenues should be explored to integrate the data generated by genetic services with the existing RD frameworks while maintaining security, privacy, and confidentiality of the patient’s health data.

### Access to treatment

The complexity in finding the right expertise for a particular RD and the affordability of the process, and the treatment, makes RD healthcare largely inaccessible to Indian patients. While the existing programs can potentially help the patients be referred to the right expertise with ease and affordably, treatment and management of RDs would still bear a substantial financial cost.

As discussed previously, only a subset of Group 1 RDs has curative treatment. NPRD 2021 provisions a one-time help of Rs 2 million for Group 1 diseases, which may be insufficient for cases requiring long-term postoperative care and management. The RBSK and child health component of the JSSK program can be expanded to cater to at least a few of such Group 1 RDs. To further expand RBSK for follow-up and postoperative management costs, possibilities should be explored. Besides the one-time help provided by the central government, aids that the state government can provide may also be looked into. Many Group 2 RDs can also be catered to by expanding RBSK and child health nutrition supplementation programs till at least up to 18 years.

However, Group 3 RDs are entirely outside the scope of the existing NHM programs. The few Group 3 RDs that have an approved drug or treatment are exorbitantly priced for Indian patients. For example, enzyme replacement therapy for Lysosomal storage disorder costs around INR 100 million a year, whereas a single dose of Zolgensma costs around 160 millions for a child [31]. The cost of these drugs puts a strain on both patients and the healthcare system. It becomes challenging to finance the treatment of these diseases from the existing budget of NHM programs. A dedicated RD management program is necessary to fund and manage these subsets of RDs.

Along the lines of New Drug and Clinical trial Rule 2019, which provided much needed incentives for orphan drugs [32], the government should further encourage private enterprises and startups to invest in development of RD drug, diagnostics and other products. These additional incentives could include secured market, tax benefits, government procurement mechanism etc. Not only the locally procured technology would lower the RD management costs for the Indian patients, but also help the industry sector to invest in RDs. Our previous study on the Indian Orphan Medicinal Product Organization reveals the immense potential of these sectors in leveraging RD management [33].

### Unified surveillance system

In India, surveillance is done through various programs running in siloes, and the data generated is not efficiently integrated. For example, the linking of mortality and morbidity data from maternal, neonatal, and child death surveillance is not yet fully integrated. However, for a heterogeneous group of disorders as RDs, it is relevant to combine these multiple programs screening for various conditions at different life stages. Therefore, in a robust healthcare system, surveillance needs to focus on real-time data capture from existing various health records and integrated via a unique health identifier. The National Digital Health Mission is in the process of implementing such a system that integrates various health data across the country [34]. There have been efforts previously through the RCH program to survey maternal and neonatal deaths to identify causal factors to inform healthcare services and policies to develop potential solutions to prevent future deaths. However, vital life data needs to be linked with morbidity and beyond infancy for more comprehensive coverage using digital solutions such as the Integrated Health Information Platform [34]. This would help identify any prevalent patterns of neonatal/child mortality and morbidity in specific communities that can be further assessed for an underlying genetic cause to elucidate the existence of any rare or undiagnosed disease. The birth defect reporting and monitoring systems in RBSK also provide an excellent opportunity to collect data for RDs in neonates and children. Assessment and analyses of this data may also provide an opportunity to focus on specific childhood disorders within the scope of NPRD 2021 and in particular geographic areas or communities to enable targeted genetic screening and awareness programprograms. An efficient strategy needs to be built to create a unified health surveillance system where all such RD-related data collected through various programs are linked to the National Rare disease registry.

## Conclusion

From the above discussion, it becomes evident that the existing public healthcare programs by NHM have immense potential to strengthen screening, prevention, and supportive care for RDs. However, diagnosis and treatment of RDs is a complicated process that requires multisystem involvement and complex care by several healthcare providers. Thus, post-screening and identifying the potential cases, a more focused pathway is necessary for an exact diagnosis and treatment of RDs. To enable smooth functioning, referral mechanisms between PHC, DEICs, and CoEs and awareness among medical professionals and healthcare providers need to be strengthened. Further, the inclusion of genetics in the NHM infrastructure can strengthen the RD care system. A unified health surveillance incorporating data across the above discussed NHM programs provides a unique opportunity to monitor RDs and supplement the National Rare Disease registry. In phase 2 of NHM, perhaps RD care may also be integrated into the thinking of early intervention programs with a more aggressive and systemic approach to lower the incidences of RDs. At the same time, avenues to an extension to RD treatment pathways may be explored to ensure universal health coverage for all.

### Limitations of the study

- The study did not conduct qualitative or quantitative research to assess the real-world challenges in adopting the NHM programs for RD care. However, the authors based their discussion on the relevant published literature.
- Health is a state subject in India, and every state/UT can adopt the National health programs based on their own needs and requirements. This study, however, is strictly limited to the National Health Mission’s programs as rolled out by the central government, and we have not explored and discussed the variations adopted in each state/UT.
- The discussion does not consider whether or not the programs have been successfully implemented. It is primarily based on the official policy or program document.

## Supplementary Information

**Additional file 1. **Extension of Table 1; table with RD strategies, public health components, NHM RMNCH+A programs, the existing public health framework and expandibility to rare diseases.

**Additional file 2. **Translation and description of India-specific terminologies.

**Additional file 3.** Description and discussion of the above mentioned NHM RMNCH+A programs, and National Digital Health Mission and UMMID program.

## Data Availability

Data sharing not applicable to this article as no datasets were generated or analysed during the current study.

## Abbreviations

ASHA: Accredited Social Health Activist
CoE: Centres of Excellence
DEIC: District Early Intervention Centres
HBNC: Home Based Newborn Care
HBYC: Home-Based Care for Young Child
JSSK: Janani Shishu Suraksha Karyakram
JSY: Janani Suraksha Yojana
MCTS: Mother and Child Tracking System
MDSR: Maternal Death Surveillance and Response
MoHFW: Ministry of Health and Family Welfare
NHM: National Health Mission
NPRD: National Policy for Rare Diseases
PHC: Primary Healthcare Centre
PHP: Primary Healthcare Providers
PMSMA: Pradhan Mantri Surakshit Matritva Abhiyan
RBSK: Rashtriya Bal Swasthya Karyakram
RCH: National Reproductive and Child Health
RD: Rare diseases
RMNCH + A: Reproductive, Maternal, Newborn, Child, and Adolescent health
SNCU: Special Newborn Care Unit
UMMID: Unique Methods of Management and Treatment of Inherited Disorders
UT: Union Territory or Union Territories
