# Supplementary material for: Integrating rare disease management in public health programs in India: exploring the potential of National Health Mission
Source: Orphanet J Rare Dis. 2022 Feb 10;17:43. doi: 10.1186/s13023-022-02194-z (PMC8832777; doi:10.1186/s13023-022-02194-z)
Supplement: Supplementary file 1 — Additional file 1. Extension of Table 1; table with RD strategies, public health components, NHM RMNCH+A programs, the existing public health framework and expandibility to rare diseases. [file 13023_2022_2194_MOESM1_ESM.docx]

## Supplementary I

**Table S1. :** A list of National Health Mission’s RMNCH+A programs that aid RDs through an existing public health ecosystem and can be expanded to cater RDs

| **RD Management strategies** | **Public Health Components** | **NHM Programs** | **Existing Public Health framework** | **Expandability to RDs** |  |
| --- | --- | --- | --- | --- | --- |
| Primary Prevention | Reproductive health | Family planning | - Counselling is provided to couples and pregnant mothers on various aspects, including birth preparedness. - Pregnancy detection kits are distributed freely by ASHA. | - Early detection of pregnancy should be promoted in families with a history of RDs, in order to register the pregnancy and for the family to avail timely and quality antenatal care under different maternal health programs. - Such pregnancies may be identified as high-risk pregnancies and should be given special antenatal care provisions and treatment management under the PMSMA program. |  |
|  | Maternal health | Janani Shishu Suraksha Karyakram (JSSK)/Janani Suraksha Yojana  (JSY) | - To register pregnancies within the system for quality antenatal care, institutional delivery, postnatal care and newborn screening, initiatives have been undertaken to reduce or eliminate out-of-pocket expenses for the mothers and children. - Pregnant mothers are provided with supplementary nutrition in their diet during pregnancy, checked for health complications and infections. Such measures reduce some of the known environmental risk factors that could cause RD in a child. |  |  |
|  |  | Pradhan Mantri Surakshit Matritva Abhiyan (PMSMA) | - Pregnancies classified as high-risk have a provision for special antenatal services, which include monitoring the birth for structural anomalies during the gestational period. Early mother-fetal interventions are provided and access to safe abortion services, if required. |  |  |
| Secondary Prevention | Newborn health | Rashtriya Bal Suraksha Karyakram (RBSK) | - Newborn screening is undertaken at all government hospitals. This involves recording vital signs and a thorough head-to-toe physical examination. | - The diseases panel for newborn and child health screening may be expanded to include diseases categorized as RDs in NPRD 2021. - Special focus should be given to children discharged from SNCUs during the home visits by ASHAs for screening for developmental delays or disabilities. - An exact diagnosis for several RDs requires specialty healthcare. Therefore, it is imperative to do a nationwide mapping of secondary/tertiary healthcare services and resources. The directory of healthcare services should be made accessible to DEICs and PHPs. |  |
|  | Child health | Rashtriya Bal Suraksha Karyakram (RBSK) | - Children aged 6 weeks to 18 years are monitored for 4Ds (Defects at birth, Deficiencies, Diseases, Development delays including disability) by mobile health teams. |  |  |
|  |  | Home-Based Young Care (HBYC) | - ASHA workers are incentivized to visit newborns till 15 months from their birth and assess the children for nutrition, health and developmental delays. |  |  |
| Diagnosis | Newborn health | Rashtriya Bal Suraksha Karyakram (RBSK) | - - Children screened for 4Ds are referred to DEICs. DEICs are the first referral points for diagnosis and management. DEICs can provide referral linkages to secondary/tertiary healthcare services. |  |  |
|  | Child health |  |  |  |  |
| Treatment | Maternal health | Janani Shishu Suraksha Karyakram (JSSK) | - Newborns are entitled to receive free immunization. - Children can receive treatment and drugs for free till the first 30 days of their birth. - In cases of emergency, transport is made freely available from the delivery point to tertiary healthcare services and back. | - The diseases panel for treatment of newborns and children under different NHM programs can be expanded to include diseases categorized in Group 1 and Group 2 in NPRD 2021. - Proper inter-state referral mechanism should be put in place for RD patients that allows them to avail treatment from tertiary healthcare service in a different state/UT or from a private partner. - Telemedicine facilities should be developed for RD patients. |  |
|  | Newborn health | Facility-based newborn and childcare units | - Newborns identified with a condition that requires urgent and immediate critical care, such as respiratory distress, are taken to neonate Intensive care units or newborn stabilization units. - Newborns and babies (< 28 days) identified with a condition that requires critical care, such as low birth weight or sepsis, but not assisted ventilation or major surgeries, are referred to SNCUs for further treatment before being referred to higher healthcare services, if required. |  |  |
|  |  | Rashtriya Bal Suraksha Karyakram (RRBSK) | - Newborns identified with either (i) neural tube defects (ii) cleft lips and palate (iii) down syndrome (iv) congenital cataract (v) congenital deafness (vi) congenital heart disease (vii) developmental dysplasia (viii) club foot (ix) retinopathy of prematurity, are eligible for free treatment and corrective surgery (wherever possible) under RBSK program |  |  |
|  | Child health |  | - Children identified with either of the conditions categorized under 4Ds in the RBSK panel are referred to DEICs for treatment and management. DEICs can further refer the patients to tertiary healthcare services, if required. The treatment is available for free. |  |  |
| Rehabilitation | Child health | Rashtriya Bal Suraksha Karyakram (RBSK) | - The patients screened and diagnosed with conditions in the RBSK panel are eligible to receive education under *Sarva Shiksha Abhiyaan*, disability scholarship and pension. - The families of the patient suffering from lifelong conditions receive counselling. | - The panel of diseases in the newborn and child health screening NHM program can be expanded to include diseases in NPRD 2021. Henceforth, patients screened and identified should also be made eligible to avail education under *Sarva Shiksha Abhiyaan* and receive disability pension and scholarship. - Counseling for the patients and their family is required to provide them support and destigmatize RDs. |  |
| Data Collection and Surveillance | Maternal health | MDSR/MCTS/RCH portals | - Mother-infant data is recorded at all institutional delivery points | - Data from various programs should be collected that is comprehensive, interoperable, compatible and maintains patients’ confidentiality. This data should be used to supplement the National Rare Diseases registry for further analyses. |  |
|  | Newborn health | Rashtriya Bal Suraksha Karyakram (RBSK)/SNCU | - Data is recorded for newborn and child health, till the age of 18, under various programs. |  |  |
|  | Child health | Rashtriya Bal Suraksha Karyakram (RBSK) |  |  |  |
|  |  | Home-Based Young Care (HBYC) |  |  |  |
