# Supplementary material for: Integrating rare disease management in public health programs in India: exploring the potential of National Health Mission
Source: Orphanet J Rare Dis. 2022 Feb 10;17:43. doi: 10.1186/s13023-022-02194-z (PMC8832777; doi:10.1186/s13023-022-02194-z)
Supplement: Supplementary file 2 — Additional file 2. Translation and description of India-specific terminologies. [file 13023_2022_2194_MOESM2_ESM.pdf]

# Supplementary II

| Terminology | Translation in English (if in Indian languages) | Notes |
|---|---|---|
| Anganwadi | Courtyard shelter | Anganwadi centres are an integral part of the Indian public health system and typically provide basic child health services in rural areas. These services include providing supplementary nutrition, health education and counselling, immunisation, and health check-ups. |
| Aspirational districts | | India ranks low on UNDP's Human Development Index (2016) due to high heterogeneity in living standards. A huge inter-state and inter-district variation was observed. Across the country, 115 districts were recognised whose upliftment in achieving key social outcomes would improve the country's rank in Human development Index. These districts have been called 'aspirational districts' and focused programs have been undertaken by the Government of India under the 'Transformation of Aspirational Districts' to improve living standards in these districts. |
| Janani Shishu Suraksha Karyakram | Mother Infant Protection Programme | An initiative under the National health Mission, this programme aims to increase institutionalised delivery by eliminating out-of-pocket expenses and providing entitlements to mothers that include antenatal, intranatal and postnatal care. Entitlements for free diagnostics, drugs, treatment and transport to tertiary healthcare services are also included for children till 30 days after they are born. |
| Janani Suraksha Yojana | Mother Protection Plan | An initiative under the National health Mission, this programme aims to increase institutionalised delivery by incentivization through cash entitlements to pregnant mothers. However, no free entitlements such as diagnostics or consumables are provided to the mother or newborn. |
| Kendra | centre | |
| Pradhan Mantri Surakshit Matritva Abhiyan | Prime Minister's Safe Motherhood Initiative | A National Health Mission initiative that aims to detect high-risk pregnancies in the 2nd and 3rd trimester for an early intervention |

| | | |
|---|---|---|
| Rashtriya Bal Swasthya Karyakram | National Child Health Programme | A National Health Mission programme for child health that aims for early screening, identification and intervention for at least 30 childhood conditions along 4D's: Defects at birth, Deficiencies, Diseases, Development delays including disability. |
| Sarva Shiksha Abhiyan | Universal Education Initiative | A Government of India initiative for the universalization of elementary education |
