# Supplementary material for: Integrating rare disease management in public health programs in India: exploring the potential of National Health Mission
Source: Orphanet J Rare Dis. 2022 Feb 10;17:43. doi: 10.1186/s13023-022-02194-z (PMC8832777; doi:10.1186/s13023-022-02194-z)
Supplement: Supplementary file 3 — Additional file 3. Description and discussion of the above mentioned NHM RMNCH+A programs, and National Digital Health Mission and UMMID program. [file 13023_2022_2194_MOESM3_ESM.pdf]

Supplementary III

A brief overview of the national missions and schemes listed in the paper. Please note, this is not a comprehensive list of all National Health Mission schemes or even all National Health Mission's RMNCH+A schemes.

National Health Mission

National Health Mission was launched in 2013 after subsuming two sub-missions: National Rural Health Mission (NRHM) and National Urban Health Mission (NUHM). NHM was launched with the vision to provide equitable, affordable and quality healthcare to all Indians. With this in view, the public health components that became focus of this programme are

(i) Reproductive, Maternal, Newborn, Child and Adolescent Health (RMNCH+A): Based on the concept of continuum of care, the overarching RMNCH+A strategy encompasses all interventions aimed at different life stages to reduce maternal and child mortality and morbidity and improve survival outcomes. The broad umbrella of RMNCH+A provides a platform to cater to different lifecycles and include health components such as HIV, adolescent health, family planning, etc for a more holistic approach. The strategy focuses on creating linkages between community-based services to facility-based services and ensuring linkages between different levels of the healthcare system through referrals and cross-referrals for the most optimum quality care.

(ii) Communicable Disease Control Programme: Various vertical programmes focusing on different communicable and infectious diseases were brought under NHM to facilitate development of technical guidelines and policies for the States and implement public health surveillance systems. The schemes under this programme are:
(a) National Vector Borne Disease Control Programme (NVBDCP), for assessment and management of diseases such as malaria, dengue, chikungunya, kala-azar, filariasis, and Japanese encephalitis.
(b) Revised National Tuberculosis Control Programme (RNTCP)
(c) National Leprosy Eradication Programme (NLEP)
(d) Integrated Disease Surveillance Programme (IDSP), a decentralized public health surveillance system for epidemic prone diseases to raise early warning to be able to undertake timely and effective public health actions in order to contain the epidemic.

(iii) Non-communicable Disease Control Programmes: To address the epidemics of non-communicable diseases (NCD) multiple existing programmes were consolidated and new initiatives were undertaken. The focus is on preventive and promotive strategies through behavioural and lifestyle changes, early diagnostics and intervention for common NCDs, capacity building and development of palliative and rehabilitative care centres. The common NCDs that are at the center of this programme include cancer, diabetes, cardiovascular disorders, mental health, visual impairment, and deafness. Other programmes include elderly care, burn management, tobacco control, palliative care, organ transplant, oral health, iodine deficiency, etc.

(iv) Health System Strengthening: A major objective of NHM is to provide equitable and quality care to all. To achieve this, public health standards need to be defined, adopted, and quality care standards need to be imparted and monitored. Resources and funding allocation, adopting standardized protocols and addressing skill gaps, therefore, are an essential dimension of NHM's operation. Under this programme, various schemes aimed at capacity building, systems and

infrastructure strengthening have been consolidated. In addition, to be able to provide the care affordably to the remotest areas of the country, there is also focus to develop mobile medical units and patient transport services, provide common essential drugs and diagnostics for free, and to integrate the tribal communities in the public health ecosystem.

(v) Infrastructure maintenance: Not only strengthening the national public health system, NHM also governs maintenance of infrastructure. Under this component, States get financial aid from NHM to meet salary requirements, training, upgradation of existing or construction of new infrastructure.

National Health Mission is the Central Government's primary programme to support the State governments health agenda. The Central Government, through NHM, can define broadly national health priorities and goals. The State governments have the flexibility to assess and implement state specific action plans. NHM has six financing components: NRHM-RCH Flexipool, NUHM Flexipool, Flexible pool for Communicable disease, Flexible pool for Non-communicable disease including Injury and Trauma, Infrastructure Maintenance, and Family Welfare Central Sector component. Depending on state's requirements, funds from the respective components are allocated to the State governments.

References:

1. https://nhm.gov.in/index1.php?lang=1&level=1&sublinkid=794&lid=168
2. https://nhm.gov.in/index1.php?lang=1&level=1&sublinkid=1056&lid=616
3. https://nhm.gov.in/index1.php?lang=1&level=1&sublinkid=1041&lid=614
4. https://nhm.gov.in/index1.php?lang=1&level=1&sublinkid=1213&lid=167

National Digital Health Mission

National Health Policy (NHP) 2017 envisaged a digital health ecosystem for the country that supports universal health coverage through an integrated health data platform to bridge the information gap between various stakeholders in the health ecosystem via digital solutions to improve transparency, efficiency, and patients' experience across public and private healthcare, while ensuring security and confidentiality of health data.

To accomplish this vision, the National Digital Health Mission (NDHM) was launched on the 74th Indian Independence Day, to be implemented by the National Health Authority under the Ministry of Health & Family Welfare. NDHM will be implemented in four phases and the key features of the first phase are,

(i) Health ID: an authenticated identification number to link patients to all their health data, to create a comprehensive national patient repository.
(ii) Digi-Doctor: a comprehensive repository of all medical practitioners
(iii) Health Facility Registry: a nationwide comprehensive repository of all medical facilities, including laboratories, diagnostics, primary and tertiary healthcare, across public and private health sectors.
(iv) Personal Health Records: electronic health-information of patients that they will have complete ownership of; they can manage and choose to share their data which may be accessed from multiple locations.
(v) Electronic Medical Records: a clinical data of a patient from a single facility, which is linked to the national repository through Health ID and can be shared with other healthcare providers after patient's consent.

NDHM is expected to contribute significantly to NHP 2017 and SDG goals. It is expected that NDHM will benefit the Indian healthcare system greatly by providing people accurate information on healthcare facilities and giving them choices on their medical data, providing healthcare providers across disciplines better access to accurate and comprehensive medical information on patients, better access to policymakers to relevant data, would allow easy geography- and demography-based surveillance, and would allow to monitor the effectiveness of different policies and interventions efficiently.

Family Planning

The family planning programme was first launched in India in 1952 and over time has evolved to cater to the country's requirements to achieve population stabilization, increased access to promotive and preventive reproductive health, and to reduce maternal and neonatal mortality.

The overarching scheme involves awareness campaigns through media and community-services, counselling on reproductive health and related matters relevant to different life stages (for example, safe sex counseling to adolescents or preconception counseling), awareness on different contraceptive methods, distribution of contraceptives, providing counceling and options on age of mother, spacing between births, provide limiting methods such as vasectomy or laproscopic sterilization, and establish and provide infrastructure for safe abortion. The multiple objectives of the scheme are implemented at all levels of health systems and involve participation from multiple partners including community-level workers, medical professionals, private sector and NGOs.

References:

1. https://nhm.gov.in/index1.php?lang=1&level=2&sublinkid=821&lid=222

Janani Suraksha Yojana (JSY)

Janani Suraksha Yojana (JSY; ***translation:*** *mother protection plan*) was launched in 2005 to promote institutionalized deliveries with the objective to reduce maternal and infant mortality. This scheme was later incorporated under NHM. The scheme is aimed at poor women, especially from low performing states. Eligible pregnant women are entitled to cash assistance for choosing to deliver at a government medical facility. Cash assistance is also available to pregnant women below the poverty line choosing to deliver at home. ASHA has been incentivized to promote this scheme among pregnant women at community-level.

References:

1. https://vikaspedia.in/health/nrhm/national-health-programmes-1/janani-suraksha-yojana
2. https://nhm.gov.in/index1.php?lang=1&level=3&sublinkid=841&lid=309

Janani Shishu Suraksha Karyakram (JSSK)

Janani Shishu Suraksha Karyakram (JSSK; ***translation:*** *mother infant protection programme*) was launched in 2011 and later brought under NHM's RMNCH+A umbrella to promote safe motherhood and institutionalized deliveries. Financial assistance provided under JSY to promote delivery in healthcare facilities was limited by socioeconomic factors that included high out-of-pocket expenses associated with hospitalization and transport facilities. To address this, MoHFW rolled out this major initiative to provide free and cashless services to all pregnant women and infants across the nation at both rural and urban government healthcare facilities.

Under this scheme, the facilities available to pregnant women include:
(i) free and cashless service for delivery at government facilities, including caesarean deliveries and exemption from any user charges;
(ii) free drugs and consumables during ANC, INC, and PNC upto 6 weeks after delivery;
(iii) free diagnostics, both essential and desirable due to complications, during ANC, INC, and PNC up to 6 weeks after delivery;
(iv) free diet during the hospitalization for delivery;
(v) free provision for blood in case of complications;
(vi) free transport from home to healthcare facility and back, and also to referral facilities and back in case of complications.

Under this scheme, the facilities available to sick newborn till first 30 days of their life include:
(i) free and zero expense treatment and exemption from any user charges;
(ii) free drugs for treatment if required and immunization
(iii) free diagnostics for infections, pneumonia, etc;
(iv) free provision for blood, if required;
(v) free transport from home to healthcare facility and back, and also in between referral facilities if required.

References:

1. https://vikaspedia.in/schemesall/schemes-for-women-schemesall/schemes-for-pregnancy-womens/janani-shishu-suraksha-karyakaram-jssk
2. http://nhm.gov.in/images/pdf/programmes/jssk/guidelines/guidelines_for_jssk.pdf
3. https://nhm.gov.in/index1.php?lang=1&level=3&sublinkid=842&lid=308

Pradhan Mantri Surakshit Matritva Abhiyan (PMSMA)

To further the aim of safe motherhood, PMSMA (***translation:*** *Prime Minister's safe motherhood initiative***)** was announced in 2016 under NHM. Timely detection and intervention of high risk factors during pregnancy and childbirth can prevent preventable deaths due to five known causal factors. Aiming at this, PMSMA envisaged detection and diagnosis of high risk pregnancies in the 2nd and 3rd trimester to provide timely and effective intervention. Family history and risk factors as diabetes, hypertension, infections, anemia, etc are recorded and investigated. Further special investigations are also offered, if required, for complicated cases. Ultrasound is also recommended in the 2nd and 3rd trimester. High-risk pregnancies are intervened under JSSK. Further, counseling is also provided to pregnant women on nutrition, care during pregnancy, identification of danger signs, birth preparedness, entitlements under JSY/JSSK, postnatal care, and breastfeeding. For unwanted pregnancies, counseling on safe abortion is also provided.

References:

1. https://nhm.gov.in/index1.php?lang=1&level=3&sublinkid=1308&lid=689
2. https://vikaspedia.in/health/health-campaigns/pradhan-mantri-surakshit-matritva-abhiyan

Facility Based Newborn and Child Care

To reduce infant and neonatal mortality, a thrust area under NHM is to develop infrastructure at different levels to facilitate newborn healthcare services. Following facilities have been directed to set up:

(i) Special Newborn Care Units (SNCU): A 12-20 bed unit with round the clock services by trained doctors and nurses, each district is supposed to have at least one SNCU.
(ii) Newborn Stabilization units (NBSUs): A 4 bedded unit at community health centres/first referral units with trained doctors and nurses to stabilize sick newborns.
(iii) Newborn Care Corners (NBCCs): A single bedded facility attached to the labour rooms/operation theatres to provide essential services to newborns.

Besides the specialized units such as above, NHM also focuses on development and strengthening of general paediatric facilities by issuing guidelines for District Hospitals to have comprehensive paediatric units comprising of eight subunits that include paediatric ward, outpatient facilities with facilities available for immunization and counselling, diarrhoea treatment, emergency triage and treatment facilities, ancillary (such as laboratory and imaging) and auxiliary facilities (such as kitchen and play area).

References:

1. https://nhm.gov.in/index1.php?lang=1&level=3&sublinkid=1179&lid=363

Rashtriya Bal Swasthya Karyakram (RBSK)

Rashtriya Bal Swasthya Karyakram (RBSK; ***translation:*** *national child health programme*) is a NHM scheme envisaged to reduce child mortality, prevent deaths by preventable causes, and improve the survival outcomes through incorporating the continuum of care in the design. RBSK achieves its objectives through early identification, diagnosis and intervention of children aged 0-18 years of age for 4Ds: Defects at birth, Deficiencies, Diseases, Development delays including disability. Under the 4Ds, over 30 health conditions have been selected for early investigation, but States may include more depending on their needs. These conditions are listed below in Fig.S1.

Defects at Birth	Deficiencies
1. Neural tube defect<br>2. Down's Syndrome<br>3. Cleft Lip & Palate / Cleft palate alone<br>4. Talipes (club foot)<br>5. Developmental dysplasia of the hip<br>6. Congenital cataract<br>7. Congenital deafness<br>8. Congenital heart diseases<br>9. Retinopathy of Prematurity	10. Anaemia especially Severe anaemia<br>11. Vitamin A deficiency (Bitot spot)<br>12. Vitamin D Deficiency, (Rickets)<br>13. Severe Acute Malnutrition<br>14. Goiter
**Diseases of Childhood**	**Developmental delays and Disabilities**
15. Skin conditions (Scabies, fungal infection and Eczema)<br>16. Otitis Media<br>17. Rheumatic heart disease<br>18. Reactive airway disease<br>19.Dental conditions<br>20. Convulsive disorders	21. Vision Impairment<br>22. Hearing Impairment<br>23. Neuro-motor Impairment<br>24. Motor delay<br>25. Cognitive delay<br>26. Language delay<br>27. Behavior disorder (Autism)<br>28. Learning disorder<br>29. Attention deficit hyperactivity disorder
30. Congenital Hypothyroidism, Sickle cell anemia, Beta thalassemia (Optional)	

Fig. S1: Health conditions managed under RBSK's 4D vision. Image accessed from http://nhm.gov.in/images/pdf/programmes/RBSK/For_more_information.pdf on September 17, 2021.

The beneficiaries of the scheme are children in rural areas and urban slums. The children aged 0-6 years would be managed at District Early Intervention Centres (DEIC). The health conditions of the children aged 6-18 years would be managed through public health channels, but DEIC would still be the referral linkage. Screening of newborns would take place at government medical facilities, while the others are screened at community level. Preschool children aged below 6 years of age are screened at Anganwadi centres and school children aged 6-18 years are screened at their government/government-aided schools. A mobile health team performs the screening at the community-level.

References:

1. https://nhm.gov.in/index1.php?lang=1&level=4&sublinkid=1190&lid=583
2. https://nhm.gov.in/images/pdf/programmes/RBSK/Operational_Guidelines/Operational%20Guidelines_RBSK.pdf

Home Based Newborn Care (HBNC)/ Home Based Young Care (HBYC)

A comprehensive scheme, Home Based Newborn Care (HBNC) was launched in 2011 that incentivized community health workers to follow-up on newborns for the first 42 days of their life and record vital data such as birth registration, birth weight, immunizations, and monitor health of the mother and the newborn. NHM provides guidelines for HBNC strategy, while States have the flexibility to develop their implementation and intervention programmes.

Home Based Young Care (HBYC) came about in 2018 as an extension of HBNC to achieve the aim of lowering under-5 child mortality specified in National Health Policy 2017. With the support of Anganwadi workers, ASHA has been incentivized to visit the newborns five additional times after the stipulated HBNC period, till 15 months of their birth, to assess the child in the four key domains - nutrition, health, development milestones, and child’s environment in terms of access to clean water, sanitation and hygiene. The community workers are expected to promote early initiation of breastfeeding and counsel on continuing breastfeeding till 2 years of age along with complementary diet, check for infections, ensure immunizations, and monitor developmental delays.

References:

1. https://hbnc-hbyc.nhp.gov.in/AboutUs/aboutHBYC
2. https://www.aspirationaldistricts.in/wp-content/uploads/2019/02/Home-Based-Care-for-Young-Child-Guidelines.pdf
3. https://nhsrcindia.org/sites/default/files/2021-05/Handbook%20for%20ASHA%20on%20Home%20Based%20Care%20for%20Young%20Child-English.pdf
4. https://hbnc-hbyc.nhp.gov.in/AboutUs/aboutHBNC

RCH/MCTS portal

To ensure the delivery of the entire spectrum of health benefits to pregnant mothers and children for quality care and to monitor the outcomes of interventions, it is essential to record this integrated data and make it accessible to stakeholders for data-driven policy decisions. To achieve this, MoHFW launched an innovative web-based portal in 2009, Mother and Child Tracking System (MCTS), to collect the following information:

For pregnant women,

- location details (state, district, block, address)
- personal details (name, date of birth, caste, UID)
- health provider details (ANM/ASHA, medical facility)
- pregnancy outcomes (including JSY benefits whether availed)
- PNC details
- infant details

were noted.

For infants,

- location details (state, district, block, address)
- personal details (name, date of birth, caste, UID)
- health provider details (ANM/ASHA, medical facility)
- immunization details

were noted.

From 2015 onwards, the data on MCTS has been subsumed in a phase manner under Reproductive and Child Health (RCH) portal, which is touted to be the 'augmented version of MCTS'. While MCTS was restricted to antenatal, postnatal and deliveries data, RCH is a more comprehensive portal that allows collection of data across the reproductive lifecycle of the beneficiary woman starting from registering as a couple. It allows to track couples for their contraceptive needs, in addition to monitoring the health care during pregnancy and to children (aged 0-5 years).

References:

1. https://rch.nhm.gov.in/
2. https://digitalindia.gov.in/content/mother-child-tracking-system-mcts
3. http://www.nhmmp.gov.in/WebContent/IMP_Notice/Draft_Revised_Data_Entry_User_Manual_ver_1_1-RCH.pdf

Unique Methods of Management and treatment of Inherited Disorders (UMMID)

A Government of India's initiative under the Department of Biotechnology, UMMID aims to implement screening for genetic disorders in newborns at government hospitals to achieve Universal health coverage. With the underlying concept of "Prevention is better than cure", UMMID is set to achieve the goals through - (i) creating awareness about genetic disorders in medical professionals (ii) capacity building in human genetics (iii) establishing NIDAN (National Inherited Diseases Administration) Kendras in government hospitals to provide genetic counseling, prenatal testing, genetic diagnostics and screen for blood disorders and treatable genetic disorders. The Centres of Excellence listed in NPRD 2021 have been enlisted as training and mentor institutes for training skilled clinicians under this programme. Currently, the programme has been piloted in seven aspirational districts to create and assess a prototype for outreach and capacity building. Aspirational districts is an initiative by Government of India that recognises 151 districts across 28 States which have made relatively less progress in achieving social outcomes in accordance to Sustainable Development Goals and overall development of these districts would improve India's human development index.

The seven aspirational districts in which UMMID pilot programme has been implemented are:
(i) Mewat, Haryana
(ii) Yadgir, Karnataka
(iii) Haridwar, Uttarakhand
(iv) Washim, Maharashtra

(v) Ranchi / Bokaro, Jharkhand
(vi) Shrawasti, Uttar Pradesh
(vii) Nandurbar, Maharashtra

The Centres of Excellence identified under UMMID initiative and NPRD 2021 are:
(i) Department of Medical Genetics, Sanjay Gandhi Postgraduate Institute of Medical Sciences (SGPGIMS), Lucknow, Uttar Pradesh
(ii) Division of Genetics, Department of Pediatrics, All India Institute of Medical Sciences (AIIMS), New Delhi
(iii) Genetics Unit, Department of Pediatrics, Maulana Azad Medical College (MAMC), New Delhi
(iv) Department of Clinical Genetics, Christian Medical College (CMC), Vellore, Tamil Nadu
(v) Centre for Genetic Studies and Research, The Madras Medical Mission, Chennai, Tamil Nadu
(vi) Diagnostics Division, Centre for DNA Fingerprinting and Diagnostics (CDFD), Hyderabad, Telangana
(vii) Department of Haematology, Christian Medical College (CMC), Vellore, Tamil Nadu
(viii) ICMR-National Institute of Immunohaematology (NIIH), KEM Hospital, Parel, Mumbai

NIDAN Kendras established and identified under UMMID initiative and NPRD 2021 respectively are:
(i) Lady Hardinge Medical College (LHMC), Delhi
(ii) Nizam's Institute of Medical Sciences (NIMS), Hyderabad, Telangana
(iii) All India Institute of Medical Sciences (AIIMS), Jodhpur
(iv) Army Hospital Research & Referral, Delhi
(v) Nil Ratan Sircar (NRS) Medical College and Hospital, Kolkata

References:

1. https://vikaspedia.in/health/nrhm/national-health-programmes-1/unique-methods-of-management-and-treatment-of-inherited-disorders
2. https://dbtindia.gov.in/pressrelease/inauguration-nidan-kendras-and-ummid-launch-dbt-website
